# Dynamic response analysis of concrete box subgrade under double-line heavy-haul railway

**DOI:** 10.1371/journal.pone.0340469

**Published:** 2026-01-20

**Authors:** Jinglei Liu, Yinghui Jin, Bo Zhao, Aixian Qin, Min Zhao, Jing Guo, Erjun Guo

**Affiliations:** 1 Hebei University of Architecture, Zhangjiakou, Hebei, China; 2 China Construction Eighth Engineering Division Rail Transit Construction Co. Ltd, Nanjing, China; 3 Hebei Key Laboratory of Diagnosis, Reconstruction and Anti-disaster of Civil Engineering, Zhangjiakou, Hebei, China; China University of Mining and Technology, CHINA

## Abstract

The concrete box subgrade, a novel structural form constructed from reinforced concrete to replace conventional fill subgrades, effectively addresses challenges associated with land scarcity and material shortages. A three-dimensional finite element model of the track-subgrade-foundation system was established via numerical simulation to assess the feasibility of the concrete box subgrade. Subsequently, a comparative analysis of dynamic stress, displacement, and acceleration were analysed under three operating conditions: bidirectional operation, unidirectional full load operation, and unidirectional empty load operation. The results reveal that, under moving train loads, dynamic stress is primarily concentrated within the active track zone. Along the vertical webs, dynamic stress initially increases before decreasing, peaking at 1.25 m (one-quarter of the vertical web depth) below the subgrade surface. At a depth of 3.0 m, the dynamic stress attenuation rate of the concrete box subgrade is 1.4 times that of the conventional subgrade, effectively mitigating stress transmission to the foundation. Under bidirectional operation, the maximum dynamic displacement of the concrete box subgrade is 0.203 mm, representing a 92.41% reduction compared to the conventional subgrade, demonstrating enhanced structural integrity and lateral deformation control. The maximum acceleration reaches 0.137 m·s^-2^, which is 78.86% lower than that of the conventional subgrade, indicating superior vibration mitigation.

## 1 Introduction

Heavy haul railway technology has developed rapidly worldwide and is now globally recognized as a pivotal direction in freight rail transportation [1–4]. With its exceptional freight-carrying capacity, heavy haul railways play an indispensable role in promoting integrated transport systems and socio-economic development [5–6]. By the end of 2024, China’s railway network had reached a total operational length of 162,000 km, with an annual freight volume of 5.18 billion tonnes.

Field and model tests are costly; however, some scholars regard experimental testing as a crucial method for investigating the dynamic response of railway subgrades. Mei et al. [7]and Li et al. [8] derived dynamic response patterns of subgrades under moving loads through field measurements. Leng et al. [9] constructed a full-scale model of a heavy haul railway subgrade to explore variations in dynamic stress and displacement under different axle loads. Ishikawa et al. [10] performed fixed-point and moving-wheel loading tests on a scaled track model, revealing that fixed-point loading tests are inadequate for assessing deformation effects on the subgrade. Currently, field and model tests are generally limited to external contour measurements, making it difficult to capture stress characteristics in the core loaded regions within the subgrade. With advancements in computational technology, numerical simulation has become a mainstream approach for investigating three-dimensional dynamic subgrade responses due to its accuracy and flexibility. Bao et al. [11–12] addressed the challenge of characterising three-dimensional stress distribution in subgrades under traffic loading by establishing a dynamic stress attenuation and three-dimensional stress calculation model. The model was validated under multiple scenarios and provides a novel concept, method, and technique for investigating the evolution of subgrade service performance under long-term traffic loading. Using finite element software, Cao et al. [13] evaluated the influence of subgrade elastic modulus and density on dynamic response. Their findings indicated that increased modulus and density enhanced dynamic stress and acceleration while reducing displacement. Yang et al. [14] employed a three-dimensional dynamic triaxial discrete element model to examine the effect of fine aggregate content in graded crushed stone. Results showed that increasing fines significantly enhanced internal frictional energy dissipation and deformation, thereby accelerating subgrade deterioration. Sun et al. [15] established a simulation model to analyse dynamic stress distribution in subgrade beds under a 30 t axle load, validating the model’s accuracy by comparing simulated results with field measurements. Thus, dynamic characteristics generated by large axle-load trains can be computed to prevent structural damage to the subgrade. Mei and Liu et al. [16–17], based on vehicle-track coupled dynamics theory [18], developed three-dimensional dynamic models using finite element software. Their studies verified that dynamic stress on the subgrade surface follows a normal distribution and identified a significant stress superposition effect when adjacent bogies pass through the subgrade, providing a reliable basis for analysing subgrade dynamic deformation and cumulative settlement.

Subgrade engineering constitutes a critical component of railway infrastructure. In contrast to conventional mixed passenger-freight lines, heavy-haul railways are dedicated freight routes characterised by high axle loads, large traction masses, and high throughput [19]. Track systems include both ballasted and ballastless designs [20–21]. Among these, ballasted tracks are widely adopted in the heavy-haul railway applications owing to their mature technology, ease of maintenance, and capacity to meet structural safety and stability demands under heavy loads. Conventional ballasted subgrades typically comprise quarried rock, natural gravel, or processed sand-gravel mixtures arranged in layered systems. Despite mechanical compaction, these subgrades are prone to consolidation settlement, particularly when fill quality is suboptimal. Additionally, their trapezoidal cross-sections require extensive land use, and the subgrades themselves bear significant self-weight, resulting in greater demand for maintenance and ground improvement. Harsh environmental conditions may further impair the structural integrity of conventional subgrades, leading to various forms of distress [22–23]. Consequently, more rigorous design and quality control standards have been introduced for heavy haul subgrade systems [24–25].

In response to these challenges, several innovative subgrade structures have been proposed and implemented in engineering practice. These can be categorised into three groups: enhancing fill material confinement (e.g., U-shaped subgrades [26–27] and dual-sided reinforced retaining wall subgrades [28]); replacing conventional subgrades with new structures (e.g., prefabricated box culvert subgrades [29] and Quadra-pot-shape earthworks [30]); and combining enhanced confinement with new structural systems (e.g., high-pile slab structures [31]). These innovations aim to address the shortcomings of conventional fill subgrades and enhance load-bearing performance. In this context, a novel concrete box subgrade structure [32]-constructed using cast-in-place or precast reinforced concrete-has been developed, which occupies less land, requires no traditional fill, offers high strength and integrity, exhibits substantial overall stiffness, enables rapid construction, and maintains a light structural weight. Zhu [33] developed a computational model of the concrete box subgrade to investigate the internal force and deformation characteristics under different loading conditions, analysed its natural vibration frequency, and evaluated its dynamic response parameters. Jia [34] examined the cumulative settlement of a ballast track concrete box subgrade under one million loading cycles, showing that both compressive and tensile stress amplitudes within the box beam remained below the concrete design strength and met the relevant technical standards. Yu and colleagues [35–37] focused on the settlement limit of ballast track concrete box subgrades, analysed the influence of foundation settlement on the static and dynamic characteristics from the perspectives of structural deformation and train operation, and established settlement limits under four different settlement patterns.

At present, studies on heavy-haul railway subgrades are largely confined to single-line scenarios, predominantly focusing on a single indicator, dynamic stress. However, most heavy-haul railways worldwide operate as double-line systems, whose operational mechanisms differ significantly from those of conventional and high-speed railways. For example, on the Shuohuang Railway, the up line serves as the fully down line, while the down line carries lighter loads, resulting in substantial differences in subgrade loading and dynamic response compared with ordinary or high-speed railways. Accordingly, a three-dimensional finite element model of a ballast concrete box subgrade for a double-line heavy-haul railway is established, simulating a train speed of 80 km·h ⁻ ¹ and an axle load of 30 t, with the up line on the left and the down line on the right. First, a comparative analysis is conducted against a conventional subgrade under identical loading conditions, geological settings, and design criteria to determine whether the novel structure satisfies engineering safety and functional requirements, thereby verifying its feasibility. Furthermore, by examining the differences in dynamic response mechanisms between the two structural forms, the study elucidates how the innovative design enhances mechanical performance, offering new perspectives for theoretical advancement and optimisation in subgrade engineering.

## 2 Model description

### 2.1 Finite element model

**Fig 1:**
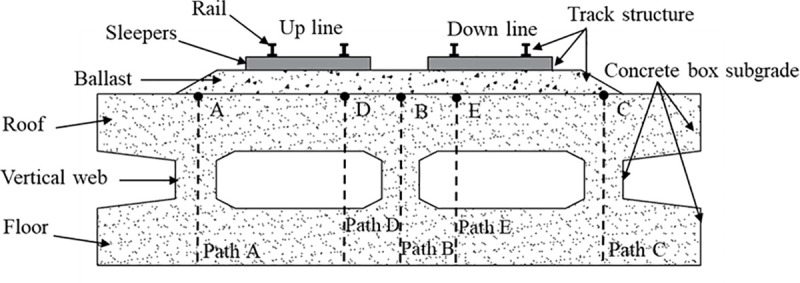
Cross-section of double-line ballasted railway with concrete box subgrade.

**Fig 2:**
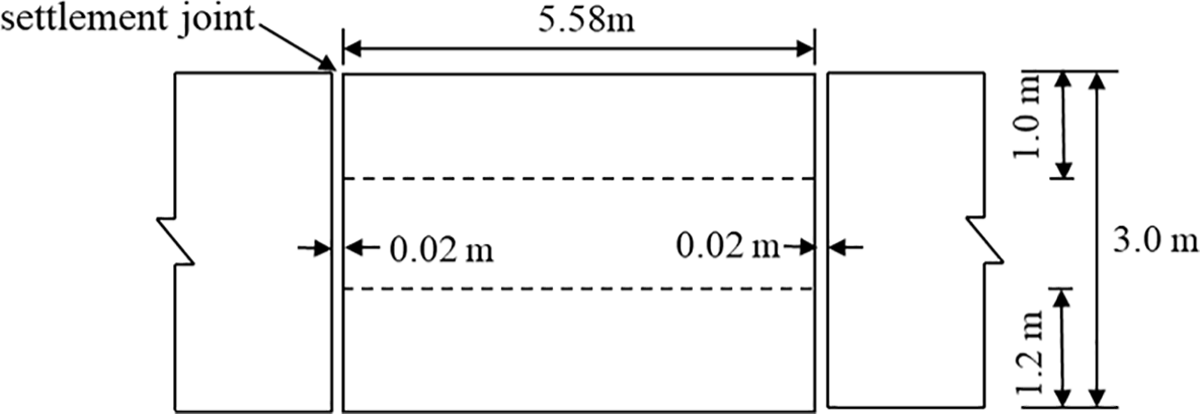
Longitudinal elevation of concrete box subgrade.

In accordance with TB 10625−2017 *Design Code for Heavy-Haul Railways* [19], a three-dimensional finite element model of the track-subgrade-foundation system was established. Fig 1 illustrates the cross-sectional layout of the double-line ballasted railway incorporating a concrete box subgrade. The track structure comprises rails, sleepers, and ballast, while the concrete box subgrade consists of a roof, vertical webs, and a floor. The left track corresponds to the up line(fully-loaded), and the right track represents the down line(empty-loaded). To analyse the dynamic response under moving heavy-haul loads, five symmetric paths along the subgrade centerline were selected. Paths A, B, and C are located at the centers of the three vertical webs, while Paths D and E are positioned directly beneath the rails. Fig 2 presents the longitudinal elevation of the concrete box subgrade, where each box unit is continuously supported by the foundation. Considering structural performance, constructability, and manufacturing feasibility, while ensuring sufficient stiffness and stability, the length of each box unit was set to 5.58 m, with a settlement joint width of 0.02 m.

**Fig 3:**
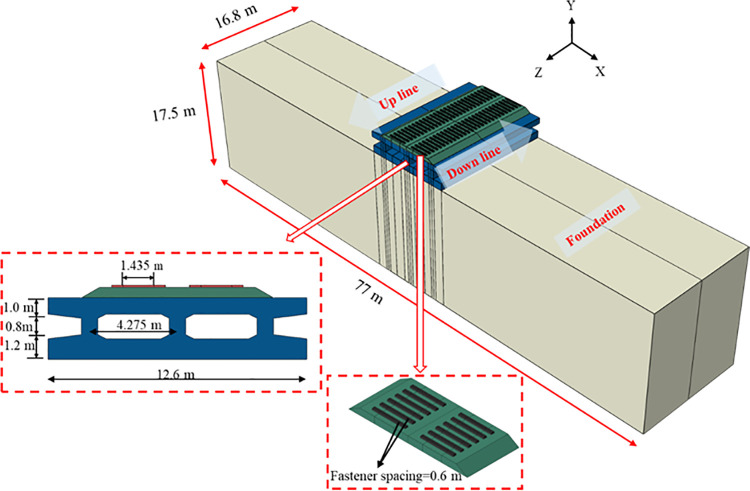
Three-dimensional finite element model.

The model dimensions were specifically designed for this study. Type III concrete sleepers were used with a simplified trapezoidal cross-section, featuring a top width of 0.2055 m, a bottom width of 0.32 m, and a height of 0.22 m. The spacing between adjacent sleepers was set at 1.435 m, with a fastener spacing of 0.6 m. The concrete box subgrade was constructed using C40 concrete reinforced with HRB400-grade steel. In the finite element model (Fig 3), the X-axis denotes the transverse direction, the Y-axis the vertical direction, and the Z-axis the longitudinal track direction. The structural dimensions of the concrete box subgrade were determined based on engineering practice. The roof spans 12.6 m horizontally with a height of 1.0 m. The vertical webs are spaced at 4.275 m intervals with a height of 0.8 m, while the floor shares the roof’s 12.6 m span and has a height of 1.2 m. The total height of the subgrade structure is 3.0 m, with a longitudinal extent of 16.8 m. To reduce stress wave reflections and improve simulation accuracy, viscoelastic boundary conditions were applied. Model dimensions directly affect both computational demand and result precision. Accordingly, the foundation domain was set to 77 m horizontal to maintain far-field boundary effects, and the vertical height was set to 17.5 m, yielding a total vertical distance of 20.5 m between the model base and the subgrade surface. This configuration meets computational requirements. Due to negligible relative slip between the ballast and the subgrade surface, as well as between the subgrade and the foundation, frictional interactions at these interfaces were neglected. Tie constraints were imposed to ensure deformation compatibility across contact interfaces [38]. All model components are represented via 8-node solid elements (C3D8R). Given the pronounced dynamic effects on the track-subgrade system under high axle-load freight train operation, both track and subgrade follow the linear elastic constitutive model. The foundation soil was represented using a linear elastic-perfectly plastic model governed by the Mohr-Coulomb failure criterion. Detailed computational parameters are summarised in Table 1.

**Table 1:** Material parameters [33,39].

Component	Bulk density (kg/m^3^)	Modulus (MPa)	Poisson’s ratio	Friction angle (°)	Cohesion (kPa)
Sleeper	2500	35000	0.2	–	–
Ballast	2400	200	0.25	–	–
Concrete box subgrade	2500	33000	0.167	–	–
Surface layer	2300	180	0.3	33	58
Bottom layer	2200	150	0.3	31	45
Layers below subgrade bed	1800	70	0.35	17	30
Foundation	1700	50	0.35	15	25

### 2.2 Viscoelastic artificial doundary

In the numerical simulation, the subgrade modelled as a semi-infinite medium. To ensure computational accuracy, viscoelastic artificial boundaries were applied at the model truncations [40–41] to simulate the absorption effect of an infinite foundation on vibration waves and to eliminate stress wave reflections that could compromise the simulation results. The theoretical formulation and implementation method for the viscoelastic boundary consider the effects of P-waves and S-waves within the plane. The stress conditions on the boundary are governed by a spring motion equation comprising a stiffness coefficient (𝜌𝑐²/2𝑟) and a damping coefficient (𝜌𝑐). By introducing appropriate springs and dampers at the truncated boundaries, the stress state at the boundary can be maintained equivalent to that of an untruncated model, thereby accurately simulating the stress conditions as scattered waves pass through the boundary.

The springs and dampers act as constraints in both the normal and tangential directions, with their parameters calculated according to Equations (2−1) and (2−2).


$$K_{BN}=\alpha_{N}G/R,\;K_{BT}=\alpha_{T}G/R$$ (2−1)

$$C_{BN}=\rho C_{P},\;C_{BT}=\rho C_{S}$$ (2−2)

Here, *K*_*BN*_ and *K*_*BT*_ represent the spring stiffness coefficients in the normal and tangential directions (N·m ⁻ ¹), respectively, while *C*_*BN*_ and *C*_*BT*_ denote the damping coefficients in the normal and tangential directions (N·s·m ⁻ ¹), respectively. *α*_*N*_ and *α*_*T*_ are the correction factors for the normal and tangential directions; based on numerical simulation results reported in references [42–44], the recommended values are *α*_*N*_ = 4/3 and *α*_*T*_ = 2/3. *R* is the distance from the vibration source to the artificial boundary point (m), *G* is the shear modulus of the medium (Pa), *ρ* is the mass density of the medium (kg·m ⁻ ^3^), and *C*_*p*_ and *C*_*s*_ are the P-wave and S-wave velocities of the medium, respectively. These parameters, calculated using the above formulas, were applied to the boundary conditions at the truncated surfaces of the heavy-haul railway concrete box subgrade model to minimise their adverse impact on the simulation accuracy.

### 2.3 Train loading

To simulate the operation of a C0-C0 type heavy-haul electric locomotive on a dedicated freight railway, an eight-car train consist was modelled with an axle load of 30 t and a running speed of 80 km·h ⁻ ¹. The primary source of vehicle-track vibrations stems from irregularities at the wheel-rail interface. To accurately capture this interaction and evaluate the subgrade’s dynamic response during train passage, a vehicle-track coupled dynamics model was employed to compute the fastener forces. To investigate the dynamic behaviour near structural discontinuities such as joints, a model capable of incorporating nonlinear material properties, detailed track geometry, structural characteristics, and discontinuities was required. Accordingly, a hybrid time-domain modelling strategy was proposed, integrating vehicle-track coupled dynamics with dynamic finite element analysis.

**Fig 4:**
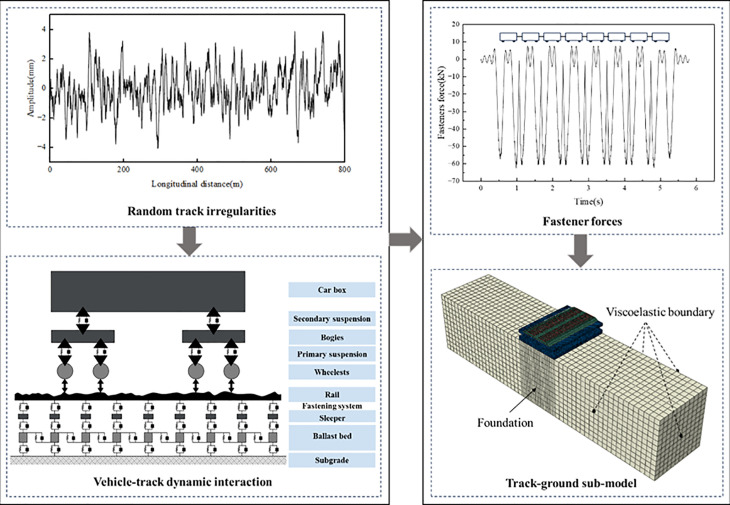
Main modelling procedure.

Based on the principles of coupled vehicle-track dynamics, an interaction model was established. A standard track irregularity spectrum for trunk lines was selected and converted into a spatial irregularity profile via inverse Fourier transform [45–46], enabling the calculation of fastener forces in the dynamic model. These forces were then applied to the three-dimensional track-subgrade-foundation finite element model. In this framework, vehicle loads were simplified as external excitations and applied at fastener positions on the sleepers, thereby simulating moving train loads and facilitating analysis of the subgrade’s dynamic response. The primary modelling procedure is illustrated in Fig 4.

## 3 Validation

As a novel subgrade structure, the concrete box subgrade has received limited attention in both theoretical research and engineering practice. Therefore, to validate the effectiveness of the numerical simulation, a conventional subgrade model was established with identical computational parameters, based on the reference data from the Shuohuang double-line heavy-haul railway, as detailed in Table 1. Subsequently, a concrete box subgrade model was developed by replacing only the subgrade structure while maintaining the same parameters to conduct the dynamic response analysis.

**Fig 5:**
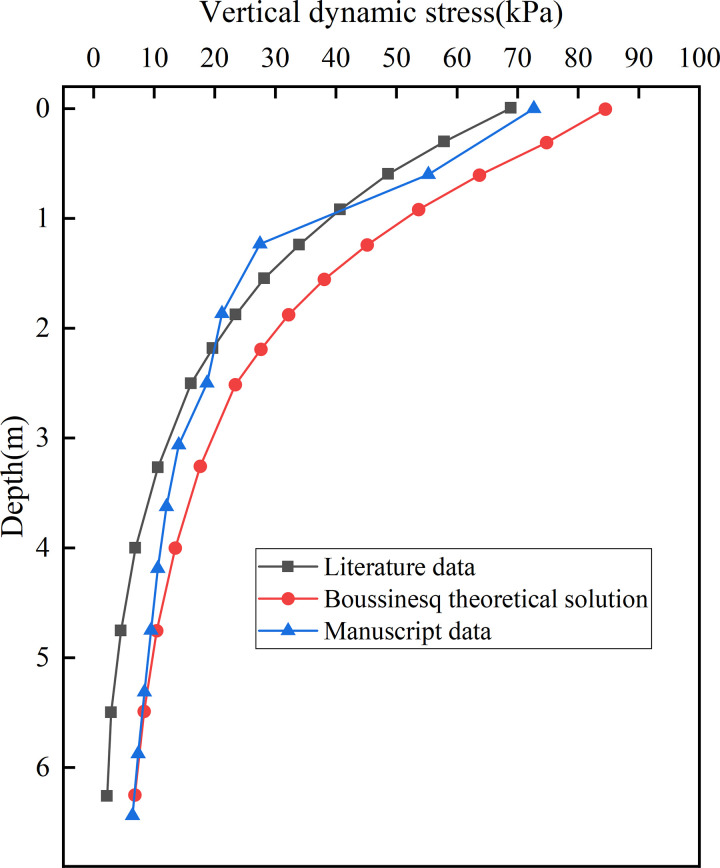
Comparison of vertical dynamic stresses in subgrade [39].

Using the conventional subgrade of the Shuohuang double-line heavy-haul railway as the background, a bidirectional operation mode with C96 open wagons was adopted. By comparing the Modelled data, it was verified that the finite element method can simulate the dynamic response of heavy haul railways across multiple dimensions with high reliability and is suitable for simulating various operational conditions. Using the finite element method, the dynamic stress directly beneath the fully loaded rail was obtained and compared with the reference data and the Boussinesq (analytical), as illustrated in Fig 5. The dynamic stress curve generated by the established model shows strong agreement with the published results, with closely matching trends.

**Fig 6:**
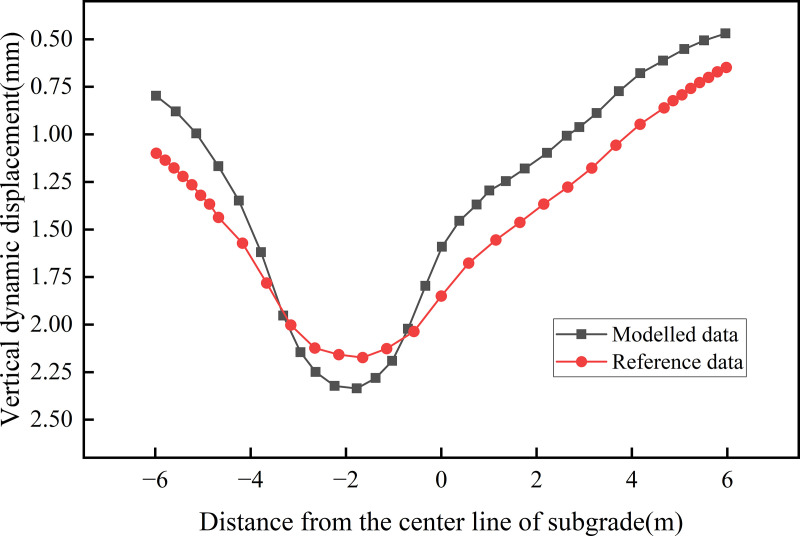
Comparison of lateral dynamic displacements in subgrade.

The vertical dynamic displacement at the subgrade surface was extracted for comparison, as shown in Fig 6. The distribution pattern of dynamic displacement along the subgrade surface is generally consistent with the reference, with the maximum calculated displacement slightly exceeding that of the reference data; however, the maximum difference is less than 0.2 mm. Some discrepancies may be attributed to the degree of mesh discretisation of the model, yet the overall distribution pattern remains well aligned.

Table 2 presents the peak accelerations at the critical locations on the subgrade of Shuohuang heavy-haul railway, including the up line center, subgrade center, and down line center. The difference between the peak accelerations of the conventional subgrade calculated in this study and the reference data is less than 1.7%. Based on this analysis, the established finite element model is considered reasonably accurate and reliable.

**Table 2:** Comparison of vertical acceleration in subgrade.

Data source	Up line center	Subgrade center	Down line center
Reference data	0.456 m·s^-2^	0.325 m·s^-2^	0.247 m·s^-2^
Modelled data	0.464 m·s^-2^	0.324 m·s^-2^	0.243 m·s^-2^

## 4 Comparative analysis and result analysis

### 4.1 Comparative analysis

Taking the double-line bidirectional operation mode with a train axle load of 30 t and a speed of 80 km·h ⁻ ¹ as an example, a comparative analysis of the dynamic responses between the conventional subgrade and the concrete box subgrade was conducted.

**Fig 7:**
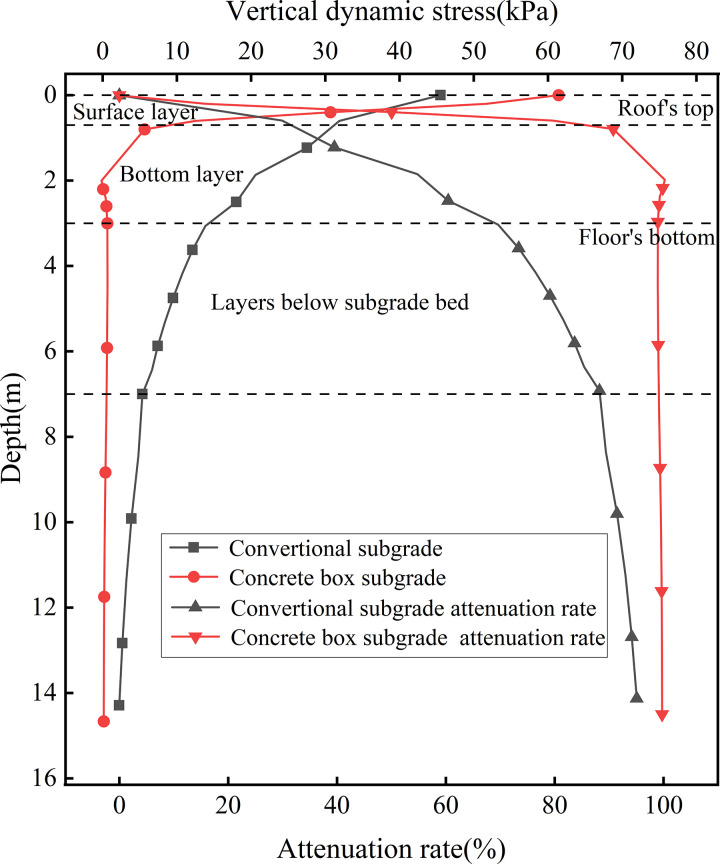
Dynamic stress distribution and attenuation curves with depth at the subgrade centerline.

Fig 7 presents the dynamic stress distribution and attenuation curves with depth at the subgrade centerline. As shown, at the subgrade surface, the dynamic stress for the concrete box subgrade and conventional subgrade is 61.43 kPa and 45.52 kPa, respectively. At a depth of 0.6 m, the stresses attenuate to 12.53 kPa and 31.91 kPa, corresponding to attenuation rates (relative to the surface stress) of 79.59% and 29.91%, respectively. At a depth of 3.0 m (the subgrade bottom), the respective attenuation rates reach 98.97% for the concrete box subgrade and 69.58% for the conventional subgrade. Within the foundation domain, the dynamic stress in the conventional subgrade continues to attenuate gradually, whereas in the concrete box subgrade it tends to stabilize at a low level. While both structures show stress decreasing with depth, attenuation is more pronounced in the concrete box subgrade. This enhanced attenuation capacity is primarily attributed to the material properties and structural from of the concrete box subgrade, which enable more effective absorption and dissipation of dynamic loads. Consequently, less energy is transmitted to the foundation, resulting in a more rapid and stable reduction in dynamic stress.

**Fig 8:**
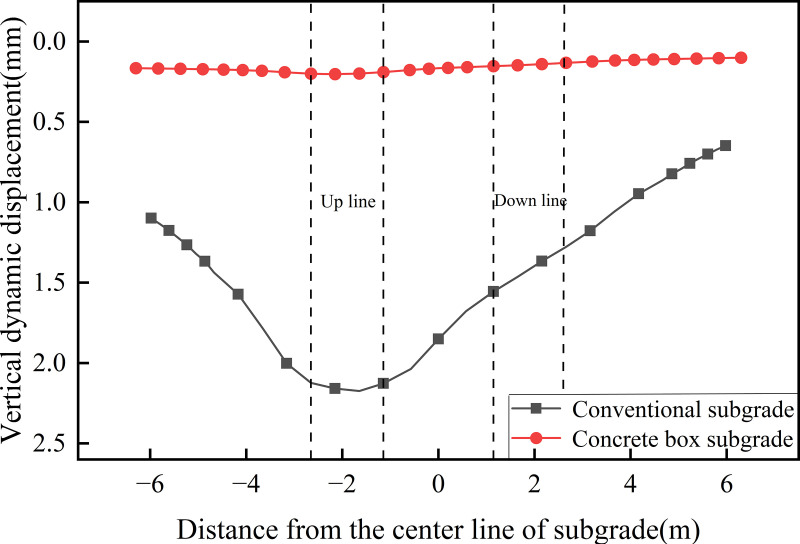
Transverse distribution of dynamic displacement along the subgrade surface.

Fig 8 illustrates the transverse distribution of dynamic displacement along the subgrade surface. As shown, the maximum surface dynamic displacement of the concrete box subgrade is 0.203 mm, located beneath the up line track; while the minimum displacement is 0.188 mm at the subgrade edge; yielding a transverse displacement variation of only 0.015 mm. In contrast, the conventional subgrade displays a characteristic “U”-shaped displacement profile, with displacement gradually decreasing from the center of the up line track towards both sides, the maximum dynamic displacement is 2.173 mm, and the minimum is 0.648 mm at the right subgrade edge, yielding a transverse variation of 1.525 mm. These results indicate that the transverse displacement amplitude of the concrete box subgrade is only 0.98% of that of the conventional subgrade, demonstrating significantly superior structural integrity. This performance is primarily attributed to the inherent stiffness of the concrete structure, which facilitates more uniform stress distribution and effectively suppresses lateral deformation under dynamic loading.

**Fig 9:**
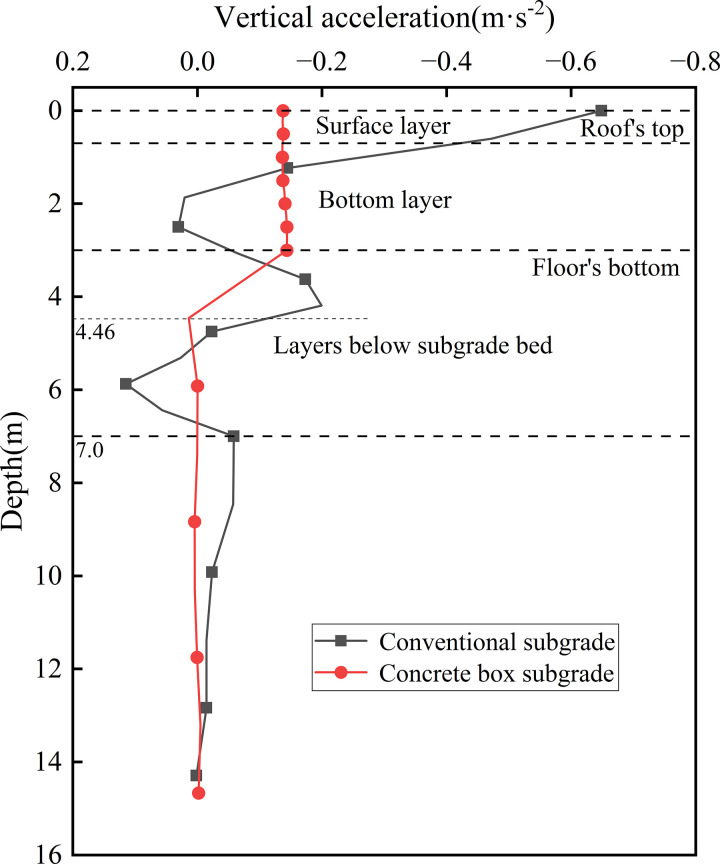
Acceleration distribution curves with depth at the subgrade centerline.

Fig 9 shows the acceleration distribution curves with depth at the subgrade centerline. As observed, the surface accelerations for the concrete box subgrade and conventional subgrade are 0.137 m·s ⁻ ² and 0.648 m·s ⁻ ², respectively, while the accelerations at the subgrade bottom are 0.143 m·s ⁻ ² and 0.064 m·s ⁻ ², respectively. For the concrete box subgrade, acceleration stabilises below a depth of 4.46 m, whereas for the conventional subgrade, stabilisation is not achieved until approximately 7.0 m. Beyond this depth, the acceleration curves for both subgrades converge and gradually decay to zero eventually. The concrete box subgrade exhibits a slower initial acceleration decay rate and a reduced fluctuation range—approximately 60% smaller than that of the conventional subgrade. This indicates that the concrete box subgrade, through leveraging material impedance differences, effectively mitigates the impact of dynamic loading and demonstrates superior dynamic stability.

### 4.2 Results analysis

The dynamic response of the concrete box subgrade under three operational conditions—bidirectional operation, unidirectional full load operation, and unidirectional empty load operation—was calculated using finite element software. Five distinct paths were selected for analysis, as illustrated in Fig 1, to investigate the distribution and propagation characteristics of dynamic stress, displacement, and acceleration within the subgrade. Specifically, Point A is located at the center of the left vertical web, Point B at the center of the subgrade (middle vertical web), Point C at the center of the right vertical web, Point D beneath the right rail of the up line, and Point E beneath the left rail of the down line.

#### 4.2.1 Dynamic stress.

**Fig 10:**
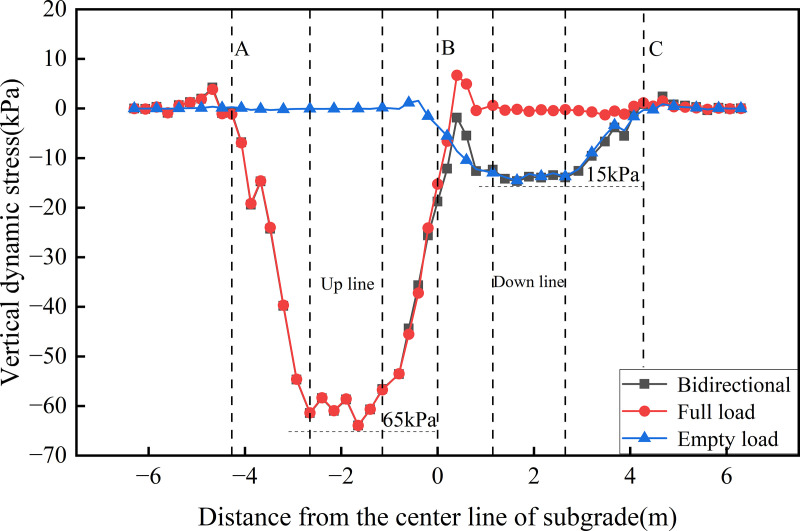
Transverse distribution of dynamic stress on the roof’s top.

**Fig 11:**
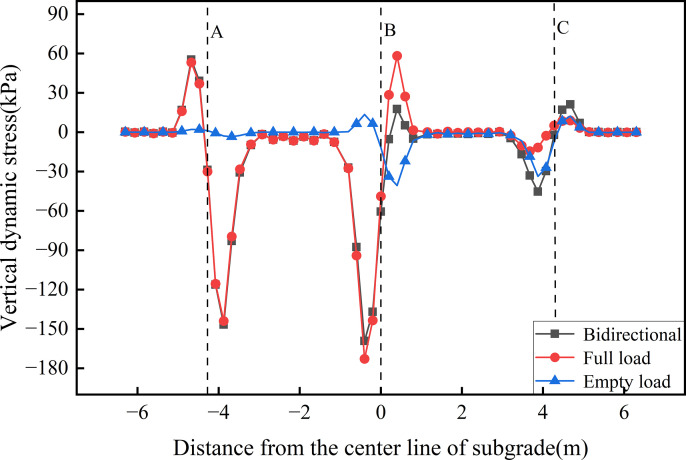
Transverse distribution of dynamic stress on the roof’s bottom.

Figs 10,11 presents the transverse distribution of dynamic stress on the roof of the concrete box subgrade under the three operational conditions. Fig 10 illustrates the stress distribution at the roof’s top. The results show that dynamic stress is primarily concentrated within the loading zones of the active tracks, with values reaching approximately 65 kPa for the up line and 15 kPa for the down line. In both bidirectional and unidirectional full load operations, two prominent stress peaks are observed at −4.675 m and 0.4 m from the subgrade centerline. These peaks correspond to zones of stress concentration induced by the train loads. This distribution pattern confirms that the concrete box subgrade effectively confines dynamic stress within the directly loaded track zones, thereby preventing excessive stress dispersion. This phenomenon is closely linked to the inherent stiffness of the concrete box subgrade structure, which provides strong integral load-bearing capacity capable of resisting load-induced stress diffusion.

Fig 11 presents the transverse distribution of dynamic stress along the roof’s bottom. The results indicate that dynamic stress is primarily concentrated at the junctions of the roof and vertical webs on either side of the rail tracks, highlighting pronounced stress concentration in these regions. Under unidirectional full load operation, the dynamic stress amplitude at the connection with the central vertical web (B) reaches −172.79 kPa (compressive stress) and 58.24 kPa (tensile stress), with the magnitude on the left side being approximately 3 times that on the right. In the bidirectional operation scenario, a clear superposition effect is observed, whereby the stress amplitude at the central web junction is slightly reduced compared with that in the unidirectional full load operation. This reduction corresponds to the dynamic stress induced under unidirectional empty load operation at the central web. The concentration of dynamic stress at the vertical web junctions along the underside of the roof highlights the critical role of these webs in load transfer and structural support. This observation provides valuable insights for the structural design of the roof-to-vertical web interface under long-term, repeated loading from heavy-haul railway traffic.

**Fig 12:**
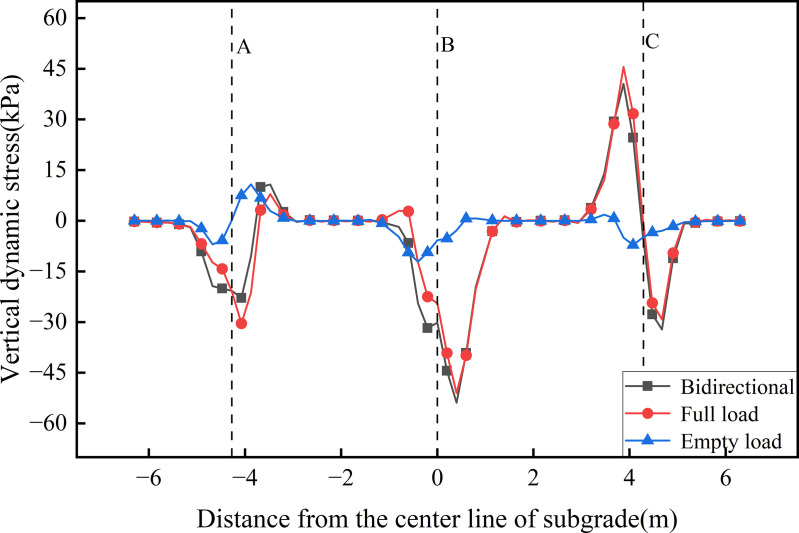
Transverse distribution of dynamic stress on the floor’s top.

**Fig 13:**
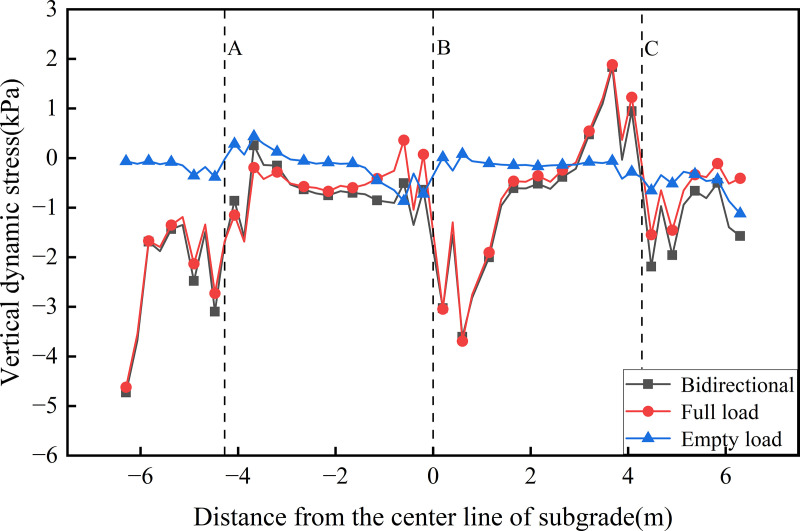
Transverse distribution of dynamic stress on the floor’s bottom.

Figs 12,13 illustrates the transverse distribution of dynamic stress on the floor of the concrete box subgrade under three operating conditions. Fig 12 presents the stress distribution on the floor’s top. Dynamic stress is primarily localised around the junctions between the floor and the vertical webs, with reduced amplitude compared to the underside of the roof’s bottom. This suggests that the vertical webs partially absorb and dissipate the transmitted stress. Under both bidirectional and unidirectional full load operations, the junctions between the left vertical web (A) and the central vertical web (B) predominantly experience compressive stress. A sharp reversal in dynamic stress (from compressive to tensile), reaching approximately 45 kPa, is observed on the left face of the right vertical web (C), indicating that full load operation induces compressive stress beneath the loaded track while simultaneously generating tensile stress on the opposite, empty-loaded side. This behaviour is attributable to the hollow configuration of the concrete box subgrade, wherein the vertical webs connect the roof and floor, facilitating internal force redistribution. Under unidirectional empty load operation, dynamic stress at the junctions between the floor and the three vertical webs remains below 15 kPa, with reverse stress emerging on the empty-loaded side. This reflects the inherent structural response of the hollow configuration under asymmetrical loading, whereby compressive stress is transferred via the vertical webs and tensile stress is induced on the opposite side. This demonstrating a cooperative load-sharing mechanism intrinsic to the concrete box subgrade. Fig 13 shows the stress distribution on the bottom of the floor. Here, the dynamic stress remains essentially stable, with a maximum amplitude below 5 kPa. This indicates that, following absorption and dispersion through the concrete box subgrade, only minimal stress is transmitted to the bottom of the floor. A slight stress dispersion effect is noted near the floor edges. The consistently low stress levels on the underside of the floor underscore the superior stress attenuation and dispersion capacity of the concrete box subgrade system.

An analysis of the dynamic stress distribution across the structural layers of the concrete box subgrade under three operational conditions reveals that stress is primarily concentrated within the loaded track zones, with pronounced concentrations occurring at the junctions of the vertical webs with the roof and floor. From the roof to the floor, dynamic stress induced by train loading is effectively attenuated through transfer and absorption by the vertical webs, as well as diffusion through the overall structure. This significantly reduces the dynamic impact on the lower subgrade components (e.g., the floor and foundation) and thereby enhances long-term structural stability. Additionally, the stress dispersion observed at the edges of the floor reflects the structure’s ability to release stress smoothly at edge zones, thereby preventing excessive concentration at the boundaries.

**Fig 14:**
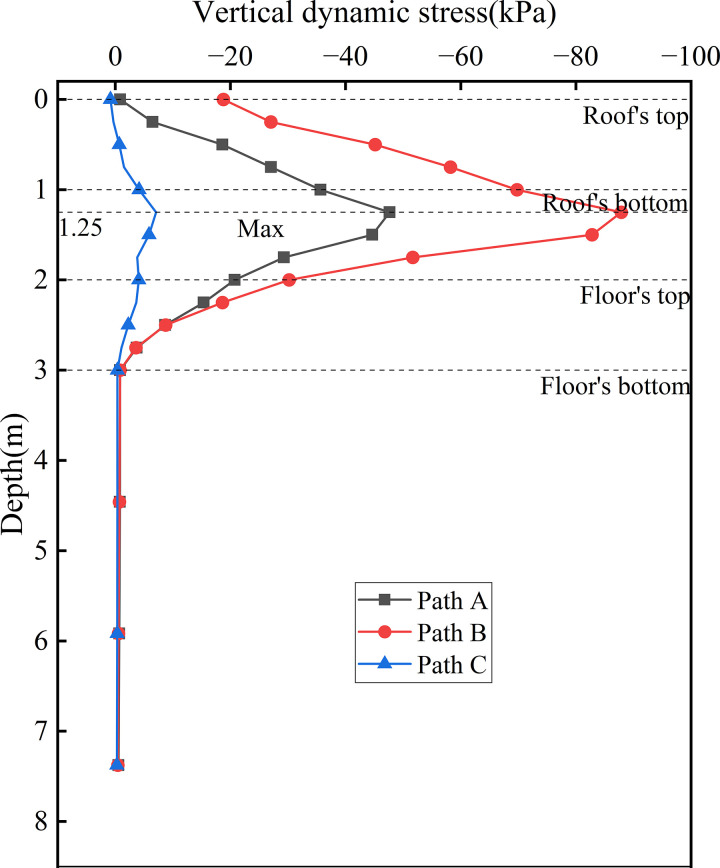
Dynamic stress distribution curves along the depth at the centers of various vertical webs.

Under the three operational modes, the bidirectional operation condition produces a relatively high level of dynamic stress across different subgrade surfaces. Therefore, the bidirectional operation mode was selected to investigate the distribution patterns of dynamic stress along the depth. Fig 14 presents the dynamic stress distribution curves along the depth at the centers of various vertical webs. The results show that, within the subgrade, dynamic stress follows a characteristic “rise-then-fall” variation pattern, peaking at a depth of 1.25 m from the surface—equivalent to one-quarter of the vertical web’s total height. Beyond this depth, the dynamic stress rapidly attenuates, falling below 1 kPa at the floor’s bottom, which demonstrates the strong stress-suppressing effect of the vertical web—floor connection. At the roof’s top, the dynamic stress at Path B reaches 18.77 kPa, while those at Paths A and C are below 1 kPa, with Path B exhibiting the most significant variation. This disparity is attributable to Path B’s central location within the subgrade. It indicating that the dynamic stresses from the tracks on both sides superimpose along the depth at the subgrade center. The observed phenomenon confirms the presence of a vertical stress superposition effect at the subgrade center under bidirectional train loading, identifying it as the core load-bearing region. With increasing depth, stress within the foundation soil gradually stabilises and approaches zero, highlighting the effectiveness of the concrete box subgrade in mitigating surface dynamic disturbances through layered absorption, multidimensional diffusion, and coordinated stress transfer.

**Fig 15:**
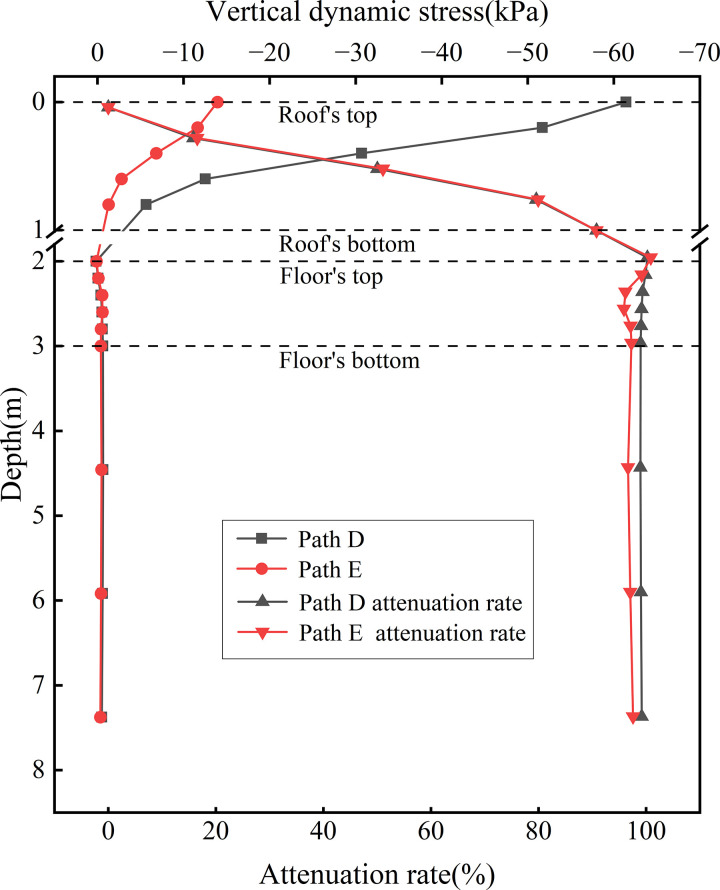
Dynamic stress distribution curves along the depth at the locations of various rail tracks.

Fig 15 presents the dynamic stress distribution curves along the depth at the locations of various rail tracks. This figure illustrates the variation in dynamic stress and corresponding attenuation rate with depth beneath the up line (Path D) and down line (Path E) rails under bidirectional operation. Due to the hollow structure of the concrete box subgrade and the absence of monitoring points between the roof and floor, the vertical axis has been truncated. on the roof of the top, the peak dynamic stress at Path D reaches 61.43 kPa, approximately 4.4 times the magnitude at Path E, highlighting not only the load disparity between fully-loaded and empty trains but also the significant influence of load magnitude on the subgrade’s stress dispersion range. The fully-loaded train induces a notably wider stress diffusion radius. Overall, both Path D and Path E exhibit a decreasing trend in dynamic stress and attenuation rate within the subgrade. The most rapid attenuation occurs within the roof, where dynamic stress reduces to −0.63 kPa and −0.75 kPa at the floor’s bottom, respectively, with attenuation rates exceeding 90%. This demonstrates the critical role of the concrete box subgrade material properties and confinement effect in initiating stress dissipation. A slight rebound in dynamic stress is observed upon entering the floor, which attributed to the interface between the floor and the foundation. At this boundary, wave reflections arise due to impedance mismatch, resulting in local stress amplification—a manifestation of dynamic interaction between the subgrade and foundation. Within the foundation soil, the attenuation rate gradually stabilises. By the bottom of the subgrade, dynamic stress has decreased by over 97%, indicating that the combined effect of the concrete box structure and foundation effectively mitigates the dynamic influence of train loading.

From the perspective of material mechanical properties, both the peak dynamic stress and internal stress differentials within the subgrade remain well below the design characteristic strength of C40 concrete (compressive strength: 40 MPa; tensile strength: 1.71 MPa). This outcome not only theoretically confirms that the concrete box subgrade structure satisfies the bearing capacity requirements, but also verifies its structural safety under long-term dynamic loading. These findings provide a solid technical foundation for future design optimisation and support the broader engineering application of the concrete box subgrade system.

#### 4.2.2 Dynamic displacement.

**Fig 16:**
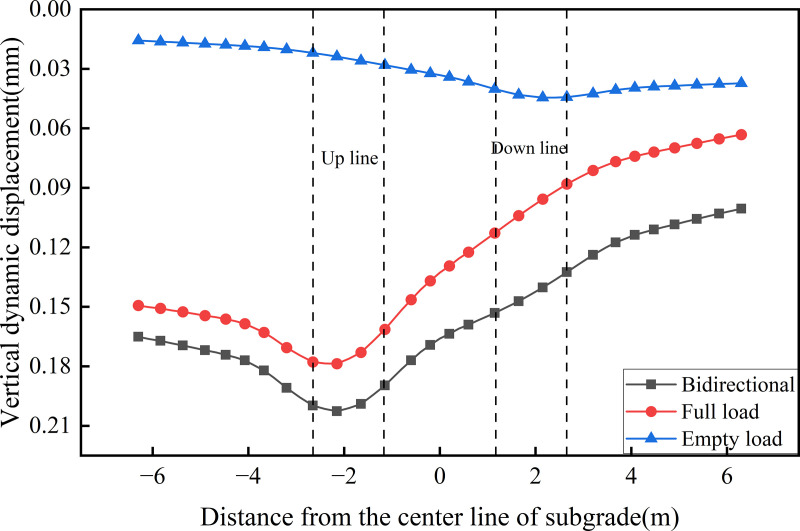
Transverse distribution of dynamic displacement on the roof’s top of the concrete box subgrade under the three operational conditions.

Fig 16 presents the transverse distribution of dynamic displacement on the roof’s top of the concrete box subgrade under the three operational conditions. As shown, the maximum dynamic displacements under fully-loaded and empty-loaded unidirectional travel are 0.179 mm and 0.045 mm, respectively, with the former being 4.01 times greater than the latter, indicating that fully loaded trains induce significantly greater subgrade deformation than empty loaded trains. Under the bidirectional operation, roof surface dynamic displacements exceed those observed under unidirectional full load operation, demonstrating a pronounced superposition effect. Specifically, the maximum dynamic displacement at the center of the up line is 0.203 mm, while that at the center of the down line is 0.144 mm, representing a 1.41-fold increase compared on the up line. In terms of maximum lateral displacement variation amplitude, the values are 0.102 mm, 0.116 mm, and 0.029 mm for the bidirectional, unidirectional full load, and unidirectional empty load operations, respectively. Overall, dynamic displacement levels remain low, reflecting the ability of the concrete box subgrade’s rigid, integrally cast structure to system effectively constrain deformation. This superior structural integrity mitigates the risk of damage associated with uneven settlement during long-term operation.

**Fig 17:**
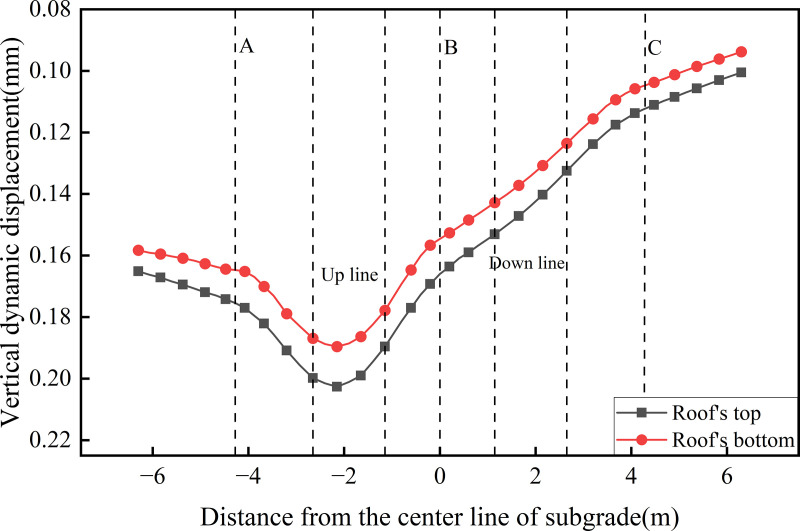
Dynamic displacement profiles along the top and bottom of the roof.

**Fig 18:**
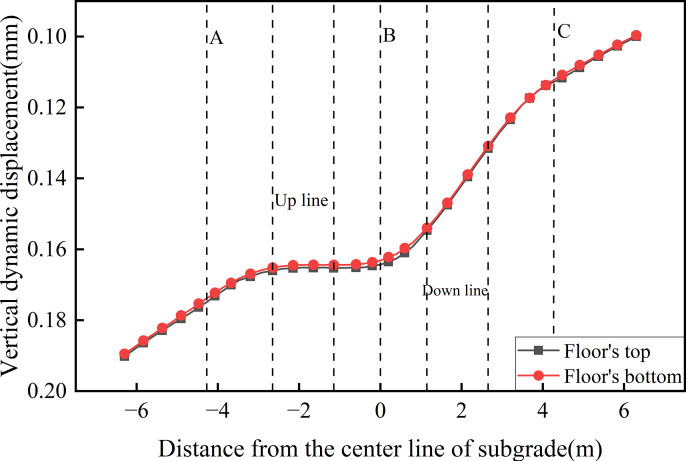
Dynamic displacement profiles along the top and bottom of the floor.

Given the pronounced superposition effect observed in surface dynamic displacement under the bidirectional operation condition, the bidirectional operation mode was selected for further investigation. Figs 17,18 presents the transverse distribution of dynamic displacement across different structural surfaces of the roof and floor of the concrete box subgrade. Fig 17 illustrates the dynamic displacement profiles along the top and bottom of the roof. Both curves exhibit similar trends, with the maximum dynamic displacement occurring at the center of the up line reaching 0.203 mm on the top surface and 0.190 mm on the bottom surface. The lateral displacement variation across the roof is 0.090 mm. Notably, the dynamic displacement on the floor remains consistently lower than that on the roof. This difference is attributed to energy dissipation during the load transfer process within the roof. The elastic deformation and internal restraint provided of the reinforced concrete absorb part of the dynamic energy, resulting in attenuated displacement transmission to the floor. This highlights the buffering capacity of the concrete box subgrade in controlling vertical deformation.

Fig 18 presents the dynamic displacement profiles along the top and bottom of the floor. As shown, the variation trends are similar and exhibit a decreasing trend from the edge of the down line track to the edge of the up line track. The maximum dynamic displacement of 0.190 mm occurs at the edge of the up line subgrade, while the minimum value of 0.100 mm is observed at the edge of the down line subgrade, yielding a lateral displacement variation of 0.090 mm. This distribution pattern is directly influenced by the hollow configuration of the concrete box subgrade structure. While the load effect from the fully-loaded train extends across a broader area, the internal hollow space beneath the rails prevents direct displacement transmission through the central region. Instead, the load is transferred through the three vertical webs. Variations in stress distribution and transmission paths across these webs result in the observed lateral gradient in floor displacement, reflecting the structural constraint imposed by the concrete box subgrade on displacement propagation pathways.

**Fig 19:**
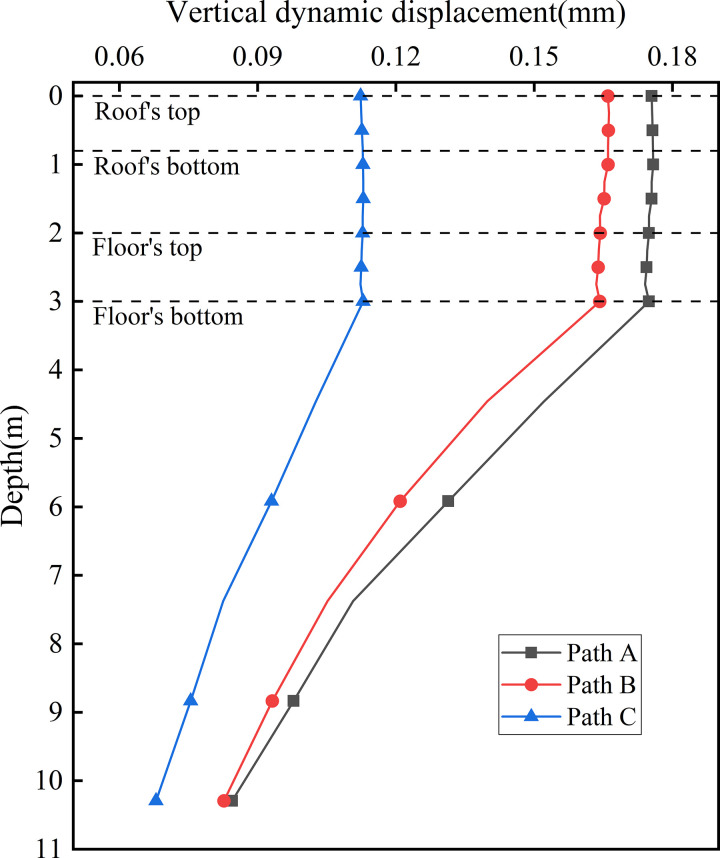
Dynamic displacement distribution curves along the depth at the centers of various vertical webs.

Fig 19 presents the dynamic displacement distribution curves along the depth at the centers of various vertical webs. The overall variation trends are similar. Within the subgrade, the peak dynamic displacements values are 0.175 mm for Path A, 0.166 mm for Path B, and 0.112 mm for Path C. Vertically, the displacement difference between Paths A and B is minor, whereas the disparity between Paths C and B is more pronounced. This indicates that the up line track (full load) exerts a stronger influence on the left and central regions, while the down line track (empty load) has a relatively weaker effect on the right side. Moreover, owing to the structural integrity of the concrete box subgrade, dynamic displacement across different structural layers along the same path remains largely uniform. With increasing depth, the dynamic displacement differences between the vertical webs gradually converge. This confirms that the concrete box subgrade facilitates uniform load transfer through integrated structural cooperation.

**Fig 20:**
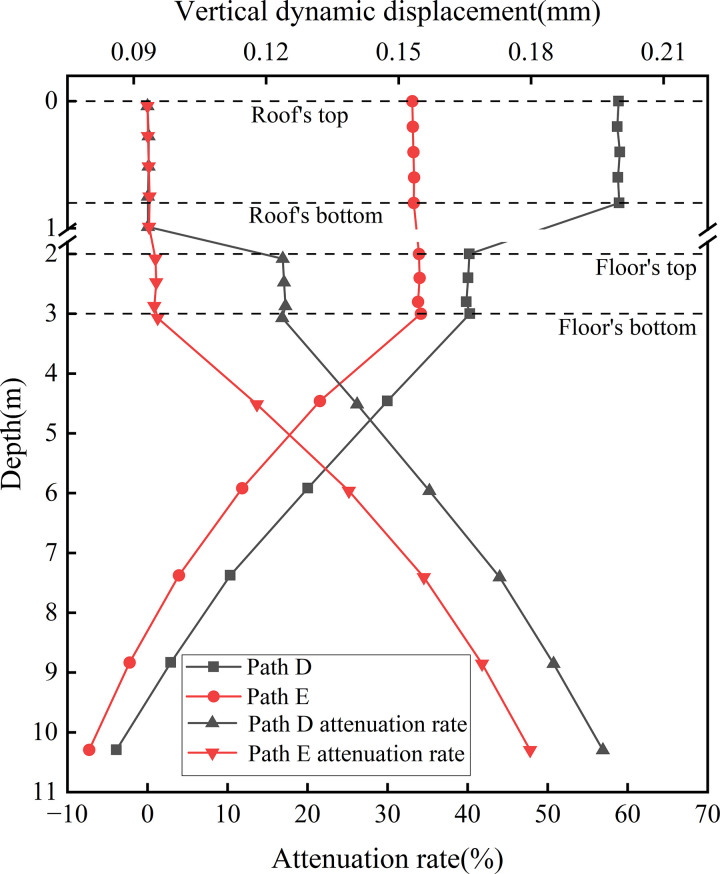
Dynamic displacement distribution curves along the depth at the locations of various rail tracks.

Fig 20 presents the dynamic displacement distribution curves along the depth at the locations of various rail tracks. This figure displays the variation and attenuation of dynamic displacement with depth beneath the up line rail (Path D, full load) and the down line rail (Path E, empty load) under bidirectional operation. For Path D, the maximum displacement at the roof reaches 0.190 mm and decreases to 0.165 mm at the floor. In contrast, Path E—subjected to lighter train loads—shows minimal displacement variation between the roof and floor, with a peak value of 0.153 mm. The dynamic displacement difference between the two paths diminishes at the floor, indicating that the concrete box subgrade effectively reduces displacement differentials through integrated deformation coordination and internal stress redistribution. This reflects the structure’s capacity for uniform deformation control. In terms of attenuation characteristics, Path D exhibits negligible attenuation within the roof and a maximum attenuation rate of 17.25% at the floor, while Path E shows almost no attenuation across the subgrade layers, highlighting that the subgrade primarily functions by transmitting loads and coordinating deformation rather than directly dissipating energy. In the foundation soil, the attenuation rate increases linearly. At 6.0 m beneath the subgrade surface, attenuation rates for Path D and Path E reach 35.23% and 25.17%, respectively; at 10.0 m, these values to 56.91% and 47.80%.

These findings indicate that the concrete box subgrade undergoes minimal internal deformation, while displacement attenuation is mainly determined by the damping capacity and plastic energy capacity of the foundation soil—validating the synergistic mechanism in which the concrete box subgrade acts as a rigid load-transmission body and the foundation soil serves as a flexible, energy-dissipating medium.

#### 4.2.3 Acceleration.

Acceleration is a critical indicator for assessing the dynamic response of the subgrade under dynamic loading. Its magnitude directly reflects the intensity of dynamic excitation on the subgrade structure: higher acceleration values typically correspond to stronger dynamic excitation, which may result in long-term deformation or even structural failure. Given the spatiotemporal transient nature of dynamic vibrations, analysing acceleration at multiple measurement points enables the identification of both its spatial and temporal distribution characteristics. Accordingly, seven primary observation points—located at the centers of the three vertical webs and directly beneath the rails—were selected for acceleration distribution analysis.

**Fig 21:**
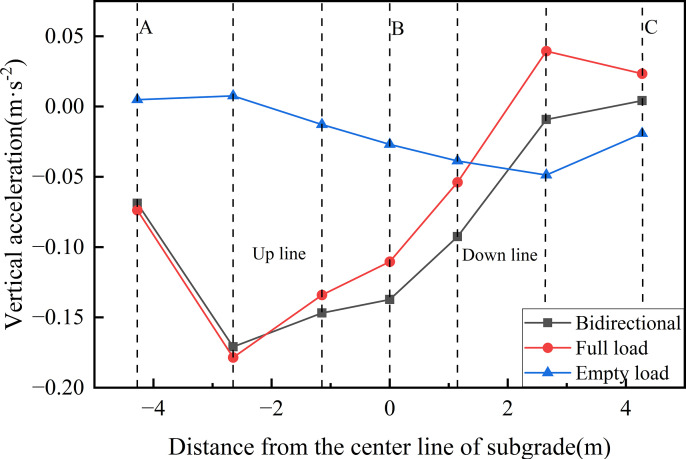
Peak acceleration curves at various measurement points on the roof surface under the three operational conditions.

Fig 21 presents the peak acceleration curves at various measurement points on the roof surface under the three operational conditions. As shown, under the bidirectional operational, the acceleration at the subgrade center reaches 0.137 m·s^-2^, which is 1.24 times and 5.1 times greater than the peak accelerations under fully-loaded and empty-loaded unidirectional travel conditions, respectively. The peak acceleration beneath the fully-loaded track is 4.1 times greater than that beneath the empty-loaded track, highlighting the combined influence of axle load magnitude on the subgrade’s vibration response. In terms of transverse distribution, under both the bidirectional and unidirectional full load operations, acceleration decreases from the up line towards the down line, whereas under unidirectional empty load travel, it decreases from the down line towards the up line. In all cases, the peak accelerations occur directly beneath the rails, confirming that train loads are transmitted through the wheel-rail interface directly into the ballast and subgrade, generating the most intense vibration response in these regions.

**Fig 22:**
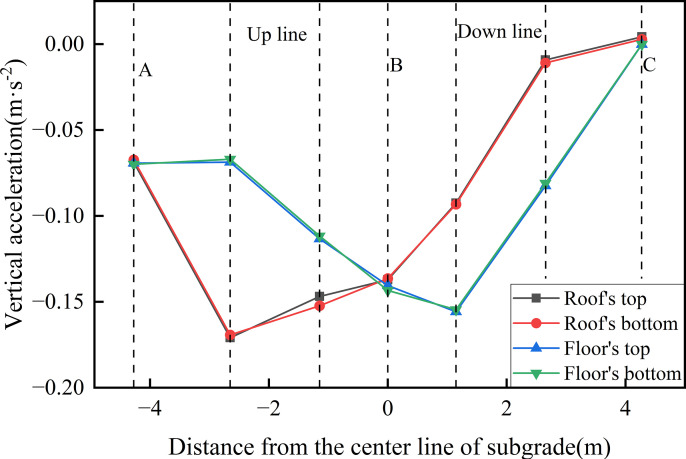
Variation distribution of peak acceleration across different structural layers of the concrete box subgrade.

Given the pronounced acceleration response observed under the bidirectional operating condition, this operating mode was selected for further investigation of acceleration distribution characteristics. Fig 22 presents the variation distribution of peak acceleration across different structural layers of the concrete box subgrade. As shown, the peak accelerations at the roof’s top and the roof’s bottom are 0.171 m·s ⁻ ² and 0.169 m·s ⁻ ², respectively, while the corresponding values for the floor’s top and bottom are 0.156 m·s ⁻ ² and 0.155 m·s ⁻ ². The negligible differences between the upper and lower surfaces of each component indicate that a high degree of structural integrity facilitates efficient lateral transmission of vibrations. The peak acceleration on the roof occurs beneath the left rail of the up line track, whereas on the floor it is located beneath the left rail of the down line track, acceleration gradually decreases towards the subgrade edges. These findings suggest that internal damping within the reinforced concrete, combined with energy dissipation at material interfaces, absorbs a portion of the vibrational energy. Moreover, the structural configuration effectively suppresses the lateral propagation of vibration waves, significantly attenuating acceleration magnitudes induced by train loading. This confirms the effectiveness of the concrete box subgrade structural design in optimising vibration control.

**Fig 23:**
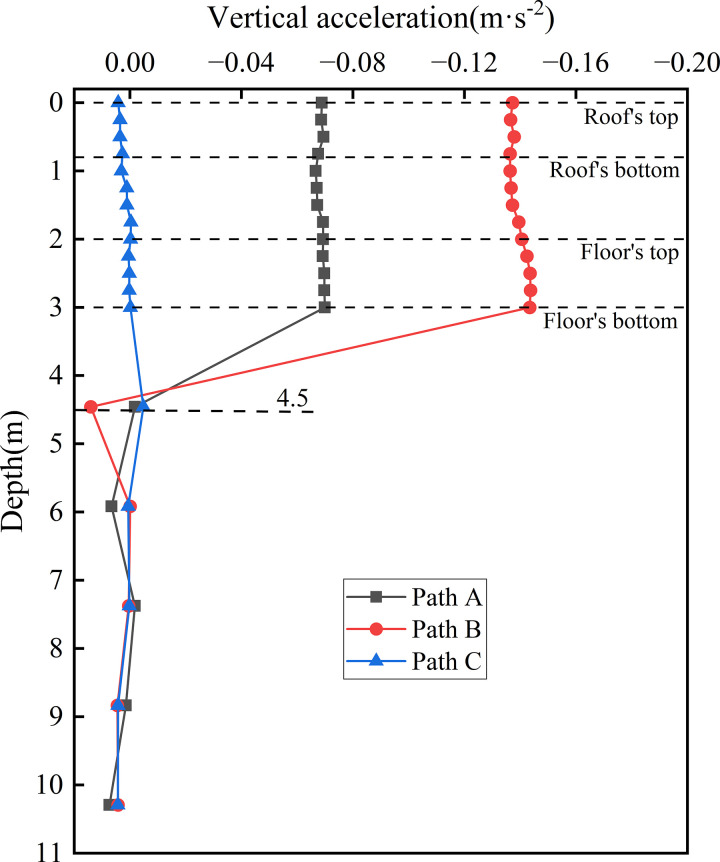
Depthwise acceleration distribution at the centers of different vertical webs.

Fig 23 presents the acceleration distribution curves along the depth at the centers of various vertical webs. This figure illustrates the variation in acceleration with depth at the centers of the left (Path A), middle (Path B), and right (Path C) vertical webs. Within the subgrade, acceleration exhibits a stabilising trend with depth. The peak acceleration values for Paths A, B, and C are 0.070 m·s ⁻ ², 0.144 m·s ⁻ ², and 0.005 m·s ⁻ ², respectively. The markedly higher acceleration at the subgrade center (Path B) reflects the concentration of vibrational energy transmitted from both the up line and down line tracks. The structural integrity of the concrete box subgrade effectively prevents local loosening or plastic deformation under moving loads, thereby avoiding vibration amplification associated with structural discontinuities or defects.

**Fig 24:**
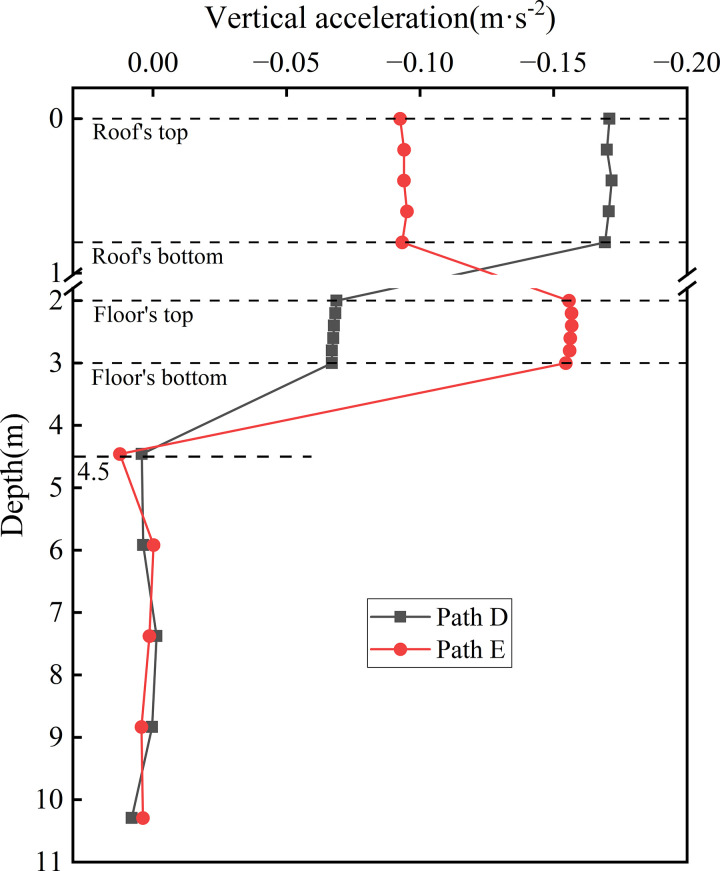
Acceleration distribution curves along the depth at the locations of various rail tracks.

Fig 24 presents the acceleration distribution curves along the depth at the locations of various rail tracks. This figure illustrates the variation in acceleration with depth beneath the fully-loaded rail (Path D) and the empty-loaded rail (Path E). For Path D, the peak acceleration at the roof is 0.172 m·s ⁻ ², decreasing to 0.068 m·s ⁻ ² at the floor. In contrast, Path E exhibits an opposite trend, with peak accelerations of 0.095 m·s^-2^ at the roof and 0.157 m·s^-2^ at the floor. This divergence arises from differences in load intensity. The fully loaded train induces stronger excitation, resulting energy dissipation and a corresponding reduction in acceleration with depth. Conversely, the weaker excitation from the empty train causes slower attenuation of vibrational energy through the rigid box structure, with minor amplification at the floor likely due to internal structural reflections.

Within the foundation soil, the acceleration curves for all paths gradually converge as depth increases, reaching maximum attenuation rate at a depth of approximately 4.5 m beneath the subgrade surface. This observation indicates that the damping characteristics and inter-particle friction, and cohesion of the foundation soil become the dominant factors governing vibration attenuation with depth. These findings further support the subgrade layered response mechanism, wherein the subgrade structure constrains the distribution of vibrations, while the foundation soil predominantly governs their attenuation.

## 5 Discussion

As an innovative structural form in heavy-haul railway engineering, the concrete box subgrade has not yet been widely adopted in practical projects. Existing studies predominantly focus on stress characteristics and settlement control for single-line ballastless tracks in high-speed railway applications, while research dedicated to double-line heavy-haul railway conditions remains limited. However, international heavy-haul railways predominantly employ double-line configurations, in which the superimposed loading effect from trains travelling in bidirectional operation can intensify structural risks. Investigating the dynamic response of concrete box subgrades under double-line heavy-haul conditions is therefore of significant engineering relevance for evaluating their operational performance.

The results indicate that the dynamic stress of the concrete box subgrade primarily concentrates within the active track loading zone. The peak dynamic stress directly beneath the fully loaded rail is substantially higher than that of the empty track, with pronounced stress concentration observed at the vertical web junctions. Meanwhile, the dynamic displacement and acceleration responses within the subgrade region remain stable, providing a valuable reference for structural optimisation. Under bidirectional operation scenarios, the superposition of dynamic loads further amplifies the dynamic responses and exerts a more significant impact on the subgrade, which warrants careful consideration in design.

This study acknowledges certain limitations in the modelling assumptions and working condition settings. First, binding constraints were applied at the ballast-subgrade and subgrade-foundation interfaces to ensure computational convergence and stability. In real engineering practice, friction exists between components, which would likely increase the peak dynamic response and reduce the propagation range of stress and vibration. Incorporating realistic friction conditions would substantially escalate the computational burden due to contact nonlinearity and will be addressed in future work. Second, the research adopts a representative heavy-haul loading scenario with an axle load of 30 t and a speed of 80 km·h ⁻ ¹, which sufficiently captures the core loading characteristics of heavy-haul railways. Since the concrete box subgrade comprises linear elastic reinforced concrete, higher axle loads would proportionally intensify the dynamic responses, whereas higher train speeds would exacerbate the responses by increasing the fastener forces, as evidenced by the comparison between fully loaded and empty tracks presented in this study. Third, the present work focuses on the instantaneous dynamic responses during train passage. The linear elastic constitutive model cannot capture long-term cumulative settlement dominated by plastic deformation or fatigue damage induced by cyclic loading. Future work will incorporate more advanced elastoplastic, damage, or viscoelastic constitutive models to enable a more comprehensive investigation of these phenomena.

## 6 Conclusions

In the context of ballasted heavy-haul railway systems, a three-dimensional track–subgrade–foundation finite element model was established, using the double-line track concrete box subgrade as the research object. The study first evaluates the feasibility of the concrete box subgrade structural design, and then conducts an in-depth analysis of its dynamic response characteristics under three train operating conditions.

Compared with the conventional subgrade, the concrete box subgrade exhibits the following structural advantages: Dynamic stress decreases progressively with depth. At 0.6 m below the subgrade surface, the stress attenuation rate increases by 49.68%, and at 3.0 m depth, the attenuation rates reach 98.97% and 69.58%, respectively, effectively reducing stress transfer to the foundation. The maximum surface dynamic displacement of the concrete box subgrade is 0.203 mm, approximately one-tenth of that of the conventional subgrade, demonstrating markedly improved structural integrity and lateral deformation control capability. The maximum acceleration is 0.137 m·s^-2^. Acceleration stabilises below 4.46 m for the concrete box subgrade, whereas stabilisation does not occur until below 7.0 m for the conventional subgrade. The fluctuation range in the concrete box subgrade is approximately 60% smaller, indicating lower acceleration response and enhanced vibration control performance.

Compared with conventional subgrades, both structural forms exhibit a decreasing trend in dynamic stress with depth. At 0.6 m below the subgrade surface, the concrete box subgrade demonstrates a 49.68% higher dynamic stress attenuation rate than the traditional subgrade. At a depth of 3.0 m, the attenuation rates reach 98.97% and 69.58% for the concrete box and conventional subgrades, respectively. The peak roof surface dynamic displacement of the concrete box subgrade is 0.203 mm—only one-tenth of that observed in the traditional subgrade. The maximum acceleration is 0.137 m·s ⁻ ², with acceleration stabilisation occurring below a depth of 4.46 m. In contrast, acceleration stabilisation in the conventional subgrade is only achieved beyond 7.0 m. The vibration fluctuation zone of the concrete box subgrade is approximately 60% narrower, indicating superior dynamic energy dissipation capacity and a lower acceleration response.

Under the bidirectional operation, dynamic stress is primarily confined within the loaded track zones, with peak values reaching approximately 65 kPa beneath the up line track and 15 kPa beneath the down line track. The peak stress beneath the fully-loaded rail is 4.3 times greater than that beneath the empty-loaded rail. Significant stress concentrations occur at the vertical web–roof/floor junctions. Vertically, dynamic stress distribution along each vertical web follows a “rise-then-fall” trend with depth, confirming the load-bearing function of the concrete box subgrade. The maximum dynamic displacement, 0.203 mm, occurs at the center of the up line track, with a maximum transverse variation of only 0.102 mm across the surface—demonstrating excellent structural uniformity. Dynamic displacement across structural layers remains largely consistent along the same track, suggesting that the primary role of the concrete box subgrade is to transmit and coordinate deformation, rather than to dissipate energy directly. The peak acceleration beneath the fully-loaded rail reaches 0.171 m·s ⁻ ², with negligible acceleration differences between the top and bottom surfaces of each structural layer. Within 1.5 m of the foundation soil, dynamic vibrational energy is significantly dissipated, affirming the layered response mechanism in which the concrete box subgrade regulates vibration distribution.

In contrast, under unidirectional operations, peak values of dynamic stress, displacement, and acceleration are concentrated on the loaded track side. Compared with the empty load condition, the full load scenario results in increases of 50 kPa in dynamic stress, 0.134 mm in displacement, and 0.049 m·s ⁻ ² in acceleration. Notably, the dynamic responses under bidirectional operation are consistently greater than those under unidirectional operation, indicating that the superposition of train loads amplifies the internal dynamic responses within the concrete box structure. This amplification may lead to increased structural damage and must therefore be carefully considered during the design process, particularly in selecting train operating modes and axle loads.

## Supporting information

S1 Data Data. (ZIP)
